# Resource constrained flux balance analysis predicts selective pressure on the global structure of metabolic networks

**DOI:** 10.1186/s12918-015-0232-5

**Published:** 2015-11-23

**Authors:** Nima Abedpour, Markus Kollmann

**Affiliations:** Mathematische Modellierung biologischer Systeme, Heinrich-Heine-Universität, Universitätsstraße 1, Düsseldorf, 40225 Germany

**Keywords:** Metabolic networks, Evolution, Bacterial metabolism, Mathematical modeling

## Abstract

**Background:**

A universal feature of metabolic networks is their hourglass or bow-tie structure on cellular level. This architecture reflects the conversion of multiple input nutrients into multiple biomass components via a small set of precursor metabolites. However, it is yet unclear to what extent this structural feature is the result of natural selection.

**Results:**

We extend flux balance analysis to account for limited cellular resources. Using this model, optimal structure of metabolic networks can be calculated for different environmental conditions. We observe a significant structural reshaping of metabolic networks for a toy-network and *E. coli* core metabolism if we increase the share of invested resources for switching between different nutrient conditions. Here, hub nodes emerge and the optimal network structure becomes bow-tie-like as a consequence of limited cellular resource constraint. We confirm this theoretical finding by comparing the reconstructed metabolic networks of bacterial species with respect to their lifestyle.

**Conclusions:**

We show that bow-tie structure can give a system-level fitness advantage to organisms that live in highly competitive and fluctuating environments. Here, limitation of cellular resources can lead to an efficiency-flexibility tradeoff where it pays off for the organism to shorten catabolic pathways if they are frequently activated and deactivated. As a consequence, generalists that shuttle between diverse environmental conditions should have a more predominant bow-tie structure than specialists that visit just a few isomorphic habitats during their life cycle.

**Electronic supplementary material:**

The online version of this article (doi:10.1186/s12918-015-0232-5) contains supplementary material, which is available to authorized users.

## Background

**Fig. 1:**
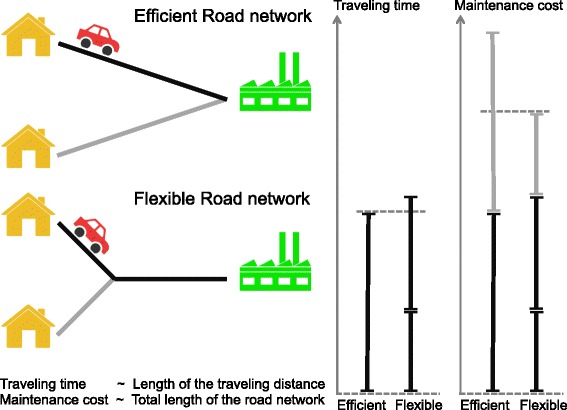
Trade-off between traveling time and road maintenance costs in road network design. Left panel: road networks that minimize either traveling time (*efficient road network*) or maintenance cost (*flexible road network*). Right panel: comparison of traveling time and maintenance cost for both road networks

Most engineered multi-task systems show a tradeoff between efficient and flexible design. A common example is the Swiss army knife, which is a flexible tool that can realize many functions but the performance of each tool function cannot reach the efficiency of specialized tools. Here the tradeoff arises from the constraint that a hand tool should not be too big to be useful, resulting in components that are shared between different tool functions. To give a more quantitative example for an efficiency-flexibility tradeoff, imagine two workers that drive between home and their common working place (Fig. 1, left panel). They both benefit from short traveling times and – as tax payers – suffer from road maintenance costs. Traveling times are minimized by direct routes from homes to working place (‘efficient’ road network). However, the road maintenance cost can be reduced if both workers share a part of the road network (‘flexible’ road network). If we assume that both benefit and cost scale linearly with road distances, the performance of the two road networks can be easily compared. The result shows that the ‘efficient’ road network has shorter driving time but higher maintenance costs than the ‘flexible’ road network (Fig. 1, right panel). By weighting traveling time and maintenance cost by a factor that reflects their relative importance, the road network with highest benefit-to-cost ratio is realized by either a more flexible or a more efficient design, depending on the weighting factor. Such a situation where one objective can only be optimized to the expense of another objective is known as Pareto optimal solution [1–3].

Due to the generality of the efficiency-flexibility tradeoff we ask whether metabolic networks show similar signatures of such a tradeoff. We define a highly flexible metabolic network by its ability to minimize the cost of up and down regulating enzymes when switching between different growth conditions. In turn, a highly efficient network is defined as one that minimizes the overall enzymatic cost under constant environmental conditions. Similar to our road network example, we expect that the catabolic pathways of a highly flexible network are shorter and converge faster to common metabolic routes. In contrast, we expect efficient metabolic networks to be more streamlined as a consequence of minimizing the overall enzymatic cost. Focusing on bacterial, our hypothesis is that species that shuttle between diverse environments show a stronger bow-tie structure than bacteria that live under more constant conditions.

For metabolic networks, a tradeoff between flexibility and efficiency can arise from limitations of cellular resources. There is currently accumulating evidence that the distribution of cellular resources is under high selective pressure, especially in fast growing prokaryotes. For example, increasing the expression of an unnecessary protein in *E. coli* leads to a linear reduction in growth rate [4, 5]. This linear relation is best explained by assuming a fixed upper bound for the protein synthesis capacity per unit biomass of a bacterial species [5].

Many attempts have been made to describe the structural features of metabolic networks [6] by considering degree distributions, small word properties [7], bow-tie-like structures [8, 9] and minimal paths between precursor metabolites [10]. Yet, it remains unclear if there exists a selective advantage that corresponds to these features. A possible evolutionary scenario is that the large-scale structure of metabolic networks is a consequence of preferential attachment, where newly acquired enzymes functionally extend the existing metabolic network structure as this can lead to an immediate fitness benefit [11, 12]. Although this principle can affect the structure of metabolic networks to significant extent, new data about the *E. coli* pan-genome showed an extreme flexibility of the gene content among closely related species by rapid acquisition of genetic material even from distantly related species due to horizontal gene transfer [13]. This observation indicates that the universe of possible metabolic networks [14, 15] can be scanned on rather short time scales and thus evolutionary innovation is not necessarily the limiting factor for structural variability. It is therefore more likely that a combination of evolutionary principles [16, 17], biochemical constraints [18], and limitations of cellular resources have shaped metabolic network structures on larger scale.

In the following, we give strong evidence that limited cellular resources in combination with fluctuating environments can lead to the observed global structure of metabolic networks. First, we formulate our assumptions within a mathematical model. Second, we illustrate the concept using a toy-model for a metabolic network structure. We show that within this simplified model, hub nodes and bow-tie structure emerge in strongly fluctuating environments. Then, we investigate the core metabolic network of *E. coli* and show that a significant structural reshaping occurs if we increase the share of investments for switching between different nutrient conditions. Finally, we investigate the large scale structural properties of more than 140 reconstructed bacterial metabolic networks. We find a highly significant correlation between the global structure of bacterial metabolic networks and their lifestyle that confirms the results of our model.

## Results and discussion

### Resource constrained flux balance model

Flux Balance Analysis (FBA) is a simple but efficient constraint-based model for cellular metabolism [19]. FBA has been successfully employed to predict genotype-phenotype relations of single gene knockout mutants [20] and the outcome of evolutionary experiments [21, 22]. The flux balance constraint ensures that the production rate of a metabolic compound equals its consumption rate at steady state. In the following we extend FBA to account for limitations of intracellular resources [23] and use this approach to simulate fluxes under fluctuating environments.

In a first step we define the universe of metabolic reactions [14, 15] as a large set of possible metabolic reactions that can be used to convert all available nutrients into biomass. An optimally adapted metabolic network for an organism can then be defined by a subset of this reaction universe that maximizes the average biomass production rate over all nutrient conditions under physicochemical constraints.

The first physicochemical constraint we account for is flux balance, which is given by

(1) $$ \sum_{i}S_{mi}{\nu^{k}_{i}}=0  $$

for each metabolite *m* (*m*=1,…,*M*). Here, *S* is the stoichiometric matrix of the universe of metabolic reactions and ${\nu ^{k}_{i}}$ is the flux of the reaction *i* (*i*=1,…,*N*) for the environmental condition *k* (*k*=1,…,*P*), where *M*, *N* and *P* are the number of metabolites, reactions and environmental conditions, respectively. Note that *S* is common for all environmental conditions and therefore does not depend on *k*.

For an enzymatic reaction, the related flux of an upregulated enzyme can be zero due to the lack of the corresponding substrate. Therefore, it is convenient to introduce the enzyme investment vector, *φ*, in addition to the flux vector, *ν*, in our model. In this manner, we separate the regulation of an enzyme and the related carrying fluxes. The relations between fluxes and investments are given by

(2) $$ -\beta_{i}{\varphi_{i}^{k}}{\le\nu_{i}^{k}}\le\alpha_{i}{\varphi_{i}^{k}}  $$

Here, ${\varphi ^{k}_{i}}$ is the enzyme investments due to the flux ${\nu ^{k}_{i}}$ and the index *i* runs over all of the enzymatic reactions plus the biomass reaction. For each enzymatic reaction, *α*_*i*_ and *β*_*i*_ are the catalytic rates of the related enzyme in forward and reverse directions, respectively, where *α*_*i*_,*β*_*i*_>0. It has been shown that the concentrations for most intracellular metabolites are much higher than the *K*_*M*_ values of the corresponding enzymatic reactions in *E. coli* [24]. This fact allows us to approximate the catalytic rates *α*_*i*_ and *β*_*i*_ as constants and thus independent of the substrate concentrations. In addition, since we consider the biomass production rate, $\nu _{i=biomass}^{k}$, as a separate reaction in our model, $\varphi _{i=biomass}^{k}$ reflects the resource investment in synthesizing biomass, where the most dominant contribution comes from the investment in ribosomes. Therefore, *α*_*i*=*biomass*_ is tightly connected to the translation rate of the ribosomes.

The environmental conditions are defined by the presence or absence of exogenous metabolites. Since metabolite concentrations do not appear in our approach, we define pseudo-reactions representing the flow of exogenous metabolites into and out of the cell environment. The bounds on the pseudo-reactions are used to express the availability of an exogenous metabolite in an environmental condition. Therefore, we consider an additional constraint, which is given by

(3) $$ {\nu_{i}^{k}}\le {u_{i}^{k}}  $$

where the index *i* only runs over the pseudo-reactions. The upper bounds, ${u_{i}^{k}}$, depend on *k* and are different in different environmental conditions. The metabolites that are not available in the environment *k* are constrained by setting the corresponding upper-bounds, ${u_{i}^{k}}$, to zero. For the available metabolites, ${u_{i}^{k}}$ is set to infinity. Note that there are no resource investment for the pseudo-reactions, since they are not real enzymatic reactions. Defining lower bounds on the pseudo-reactions is not necessary.

To generalize the resource constraint to fluctuating environments, we define two kinds of investments: metabolic investments for each environmental condition (efficiency related); and investments required for switching between different environmental conditions (flexibility related). These two contributions lead to a resource constraint that is given by

(4) $$ \sum_{i}{\varphi_{i}^{k}}+\varphi'^{k}\le\varphi_{0}  $$

where the index *i* runs over all enzymatic reactions, including the biomass reaction. Here, *φ*_0_ is the maximum capacity for cellular resources and $\sum _{i}{\varphi _{i}^{k}}$ represents the constant investment in transporters, enzymes, ribosomes and other essential parts required for growth in environment *k*. In addition, *φ*^′^^*k*^ represents the switching investments that arise from the necessity of sufficiently fast *de novo* synthesis of proteins – which implies additional allocation of ribosomes and components involved in regulating gene expression, such as transcription factors and signaling pathways [25].

Changes in nutrient conditions are in general followed by large changes in the distribution of metabolic fluxes, which in general involves the up- and downregulation of metabolic pathways [26]. The regulation of enzymes and transporters with respect to nutrient availability is likely the consequence of an efficient distribution of the protein synthesis capacity in prokaryotes. This view is supported by the experimental fact that the increase of the protein production rate for an unnecessary protein linearly decreases the growth rate [5]. It therefore makes sense to approximate the resource investments associated with switching from environment *l* to environment *k*, *φ*^′^^*k*^, by a linear function of the investment differences between these environments

(5) $$ \varphi'^{k}=\frac{r}{P-1}\sum_{l\ne k}\sum_{i}|{\varphi_{i}^{k}}-{\varphi_{i}^{l}}|\quad.  $$

Here, we assume for simplicity that all environments are visited with equal probability. The relative contribution of the resource investment associated with switching between environments are related by a coefficient *r*>0 – the switching parameter. By definition, *r* is a dimensionless factor which represents the importance of the switching investments relative to the resources invested in growth. Organisms that need to adapt their metabolism faster to a new environment, are expected to show a higher investment on resources associated with switching between environments. Evidence is provided by *E. coli*, where only five of the seven ribosomal RNA genes are needed to support near-optimal growth under constant nutrient conditions, but all seven are necessary for rapid adaptation to nutrient changes [27]. The link to the growth rate is given by the fact that ribosomal RNA genes limit the synthesis rate and thus the abundance of ribosomes.

To generalize the objective function to fluctuating environmental condition, we use a linear combination of biomass reaction fluxes with equal weight on each environmental condition

(6) $$ \max_{\nu, \varphi} \left(\frac{1}{P}\sum_{k}\nu_{i=biomass}^{k} \right).  $$

In other words, we maximize the average biomass production rate (growth rate) of the organism over all environments. Note that by maximizing this objective function, the optimal fluxes and the optimal investments are found simultaneously.

To infer a metabolic network, we solve the linear programming problem of Eqs. 1, 2, 3, 4, 5 and 6. The reactions for which the corresponding optimized investment is nonzero, at least for one environmental condition, are considered to be present in the metabolic network. The reactions for which the corresponding optimal investment is zero for all environmental conditions are superfluous and therefore are not included into the metabolic network.

### Network structure and regulation transitions

**Fig. 2:**
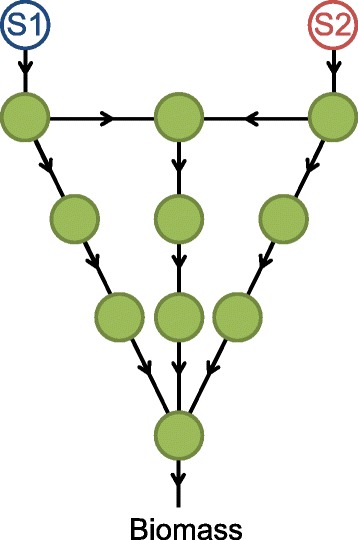
Toy-model. The universe of metabolic reactions for a simple toy-model containing 12 metabolites including two extracellular substrates S1 and S2 and 14 reactions. A fluctuating environment is generated by shuttling between the two alternative substrates, S1 and S2. The objective function is to maximize the biomass production rate averaged over all environmental conditions

To understand the concept of optimal design in metabolic networks we start with a simple toy-network as the universe of reactions, which mimics the relevant large-scale features of real metabolic networks. Consider the simple universe of metabolic reactions illustrated in Fig. 2. This directed network has 12 metabolites (nodes) including two extracellular substrates *S*1 and *S*2 and 14 enzymatic reactions (edges), which includes a biomass reaction. The first two reactions are responsible for the uptake of two alternative input metabolites *S*1 and *S*2, which we assume to be available under two different environmental conditions. The final reaction is the biomass reaction, which takes part in the objective function of our resource-constrained optimization approach. All other reactions are intermediate reactions that convert input metabolites into biomass. The catalytic rates of all reactions are equal. For each environmental conditions, an optimal network is inferred via our resource constrained FBA represented by Eqs. 1, 2, 3, 4, 5 and 6.

**Fig. 3:**
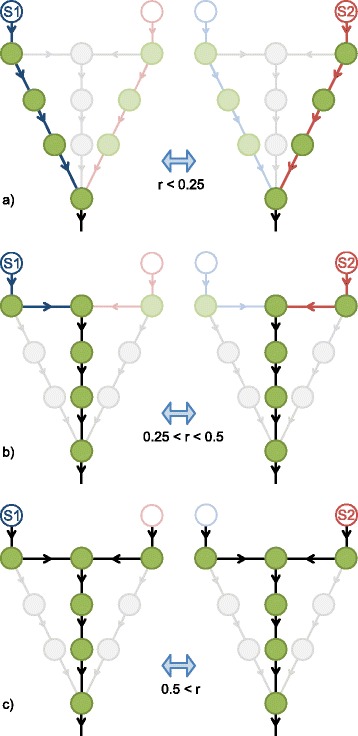
Structural transitions and regulations of the toy-model. Blue and red arrows are regulated enzymatic reactions and black arrows are constantly active enzymatic reactions. Light-gray arrows shows the remaining reactions that belong to the universe of reactions of Fig. 2, but not selected within the optimal metabolic network. Bold-lines illustrate the active reactions in each environmental condition. **a** Low switching parameters, *r*<0.25, result in an efficient network design that connects the actual present substrate to the biomass reaction using the most direct metabolic route. **b** Intermediate switching parameters, 0.25<*r*<0.5, shows a strong reduction of enzymes that are regulated and the emergence of a common permanently upregulated metabolic route. **c** High switching parameters, *r*>0.5, result in permanent upregulation of all enzymes and a network design of minimal size

We observe two different types of transition in the structure and the regulatory strategy of the optimized network by increasing the switching parameter, *r*. The first transition occurs at *r*=0.25 and shows a change in the structure together with a change in the regulation of the network. The different structures of the optimized metabolic network for *r*<0.25 and *r*>0.25 are shown in Fig. 3a and b, respectively. The two different pathways in Fig. 3b have six reactions each and are therefore longer and more costly than the two pathways in Fig. 3a, which have only five reactions. However, the cost for switching to the new environment is lower for the network in Fig. 3b with longer pathways, as it has less unshared reactions for the two different environmental conditions. The pathways in Fig. 3b have just two unshared reactions which are upregulated upon switching conditions, whereas the network of Fig. 3a has four unshared reactions to be regulated (blue and red reactions). The reactions that are shared between pathways are constantly upregulated under all environmental conditions (black reactions). Here, a tradeoff between the constant investment in enzymes and the investments in up- and down regulation of sub-pathways shapes the structure of the network. However, the strategy of regulation, which is expressing enzymes only when they are needed, does not change in this regime of switching parameters.

The second transition occurs at *r*=0.5. The nature of this transition is different from the first one, as it changes the strategy of regulation but preserves the structure (Fig. 3b and c). For *r*<0.5, the organism tries to regulate the necessary enzymes of each environment with respect to nutrient availability. However for *r*>0.5, the organism expresses all enzymes of the network constantly over time so that they can be utilized immediately in the appropriate condition. Here, the cost of preparing the cell for the new environment becomes higher than the cost of permanently synthesizing the unnecessary enzymes which can happen in really fast fluctuating environments comparable to the cell cycle timescales.

**Fig. 4:**
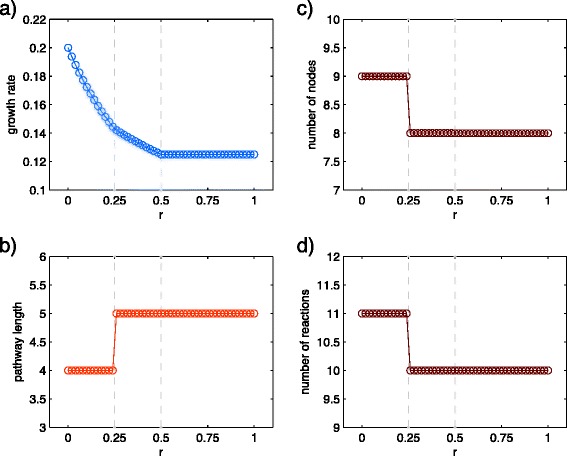
Structural parameters for the toy-model. **a** Growth rate for different switching parameters, *r*. Two discontinuities exist in the slope of the curve at *r*=0.25 and *r*=0.5. **b** Pathway length vs. *r*. **c** Number of nodes vs. *r*. **d** Number of reactions vs. *r*. Structural parameters of the optimized network confirm that only *r*=0.25 is a structural transition

As it is shown in Fig. 4, both of the transitions are reflected by two discontinuities in the slope of the growth rate as a function of *r*. The transition at *r*=0.25 is accompanied by changes in structural parameters (Fig. 4b-d). The analytic calculations of transition points are presented in (Additional file 1: Text S1).

### Emergence of the hub nodes and bow-tie structure

All currently reconstructed cellular metabolic networks show a bow-tie structure on larger scale [8], where one side of the bow-tie consists of catabolic pathways and the other side consists of anabolic pathways. The both sides converge at about twelve precursor metabolites. The precursors can be considered as hub nodes in the network, which are defined by their comparatively higher number of connections to the other nodes in the network. Under fluctuating environmental conditions with high enough switching parameter, the optimized toy-network shows similar structure where the blue and red pathways meet at the hub node and share the remaining enzymes to realize biomass production (Fig. 3b). This structure reflects the principles of a bow-tie structure by having a hub node as center part, two short catabolic pathways, and a single anabolic pathway. According to this observation our theory predicts that under fluctuating nutrient conditions with sufficiently high demand for switching cell resources and under limited cellular resource condition a bow-tie structured network emerges, which performs better than a streamlined network.

### *E. coli* core metabolic network as a universe of reactions

**Fig. 5:**
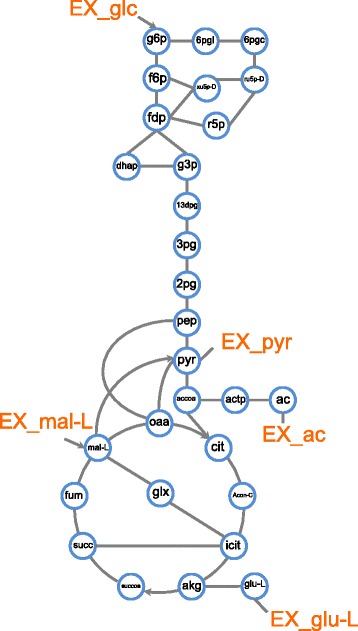
Schematic graphical representation of the *E. coli* core metabolism. The network contains 72 metabolites and 95 reactions, including pseudo reactions [29]. Some important metabolites are named explicitly. Ex_ac, Ex_pyr, Ex_mal-L, Ex_glu-L and Ex_glc represent the extracellular concentrations of Acetate, Pyruvate, L-malate, L-glutamate and D-glucose, respectively, that are provided in random order to mimic fluctuations in nutrient availability

**Fig. 6:**
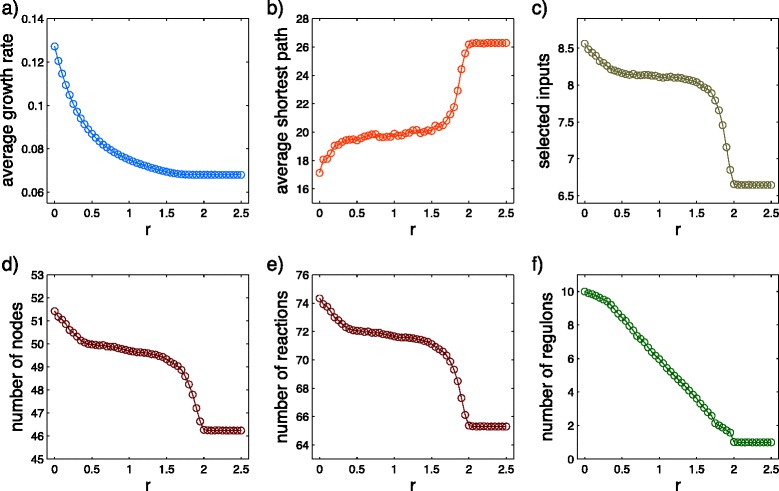
Structural parameters for the *E. coli* core metabolic network. The average over 400 random realizations of *α* and *β* with 90 percent noise is taken. Here, 5 different environmental conditions that represent different carbon sources with 2 to 6 carbon atoms is used. **a** Average growth rate vs. *r*. **b** Average shortest path from an input metabolite to each of the biomass contents for the optimized network vs. *r*. **c** Number of selected input metabolites after optimization process vs. *r*. **d** Number of nodes of the optimized network vs. *r*. **e** Number of reactions of the optimized network vs. *r*. **f** Number of regulons vs. *r*

To generalize the toy-network to real metabolic networks we would need to define a suitable universe of possible enzymatic reactions. One could imagine to include all metabolic reactions that are classified by an enzyme commission number (EC numbers). Unfortunately, the corresponding chemical reactions associated with each EC number are not clearly defined. Alternatively, we could consider all enzymatic reactions of known enzymes that are listed in databases such as the KEGG database [28]. However, as the known enzymes are already a part of the evolved bow-tie structured networks, it is unclear if alternative enzymatic routes exist that can circumvent the existing precursor metabolites. Therefore, it is not possible to define the genuine universe of metabolic reactions. However, investigating any realistic network seems beneficial to see whether the general restructuring observed in the toy-network can be observed in real metabolic networks when the switching parameter is sufficiently large. For this purpose, we consider the well characterized *E. coli* core metabolism, defined in [29], as the universe of metabolic reactions (Fig. 5). As *E. coli* is a generalist bacterium that can digest many different nutrients, its core metabolism is a collection of various important metabolic pathways that are observed in different organism. Furthermore, it has relatively small network size which simplifies the demanding numerical calculations. Due to the numerical complexity of the optimization problem, standard linear programming routines as provided by numerical packages are in general not able to find a solution for larger networks, such as the reconstructed *E. coli* metabolic network, in presence of several environmental conditions. We consider 5 different environmental conditions each containing a different carbon source as substrate which the number of carbon atoms range from two to six by using Acetate, Pyruvate, L-malate, L-glutamate and D-glucose. All of the carbon sources are considered to be metabolized under aerobic conditions under saturating concentrations of Ammonium, Phosphate and *CO*2, except D-glucose, which is metabolized under anaerobic conditions. One can use the same catalytic rates for all enzymes since different values for catalytic rates just change the position of observed transition points and do not change the qualitative behavior of transitions. However, to check the sensitivity of the results to the catalytic rate values, we compare the results for equal catalytic rates with a various catalytic rates case taken randomly from a uniform distribution with equal mean value and 10 percent of random fluctuations (see “Methods”). The results are almost the same (Additional file 1: Figure S5 and Figure S6). We also take the average over 400 realization for an extreme case with 90 percent of random fluctuations to observe the overall behavior independent from the exact values of the catalytic rates (Fig. 6). The optimal solutions are not degenerate as the resource constraint, Eq. 4, breaks the degeneracy of alternative pathways [30], if *α*_*i*_ and *β*_*i*_ have different values.

We observe a significant structural reorganization of the core metabolic network by increasing switching parameters, *r* (Fig. 6). The behavior of the structural parameters are similar to the toy-network. An obvious effect of an increase in *r* is a decrease in the growth rate (Fig. 6a). As the resource capacity of the cell is limited, an increase in the investments for switching conditions reduces the ability of the cell to dedicate resources to biomass production.

As discussed before, by choosing the *E. coli* core network we restrict the solution space to a pre-structured bow tie like universe of metabolic reactions. However, the optimally selected metabolic networks are subsets of the core network and therefore do not have necessarily similar structures. Our results show a tendency for higher switching parameters toward having more bow tie like structure. Similar to the toy-network the average pathway lengths increases while *r* increases (Fig. 6b). Here, we measure the average pathway length by the average shortest path (ASP), which is the average of the shortest routes from an input metabolites to each of the biomass metabolites. Viability ensures that each biomass content is connected to at least one input metabolite. An increase of the ASP shows the transformation of the network structure from having short direct pathways to having longer but partially shared pathways.

The number of regulons offers a simple measure to monitor strategic changes in the metabolic regulation. Here, regulons are the set of metabolic genes that are regulated in concert. Therefore, it is reasonable to assume that genes that are regulated together under different environmental conditions are a part of the same regulon. In Fig. 6f, the number of regulons varies for different switching parameters *r*, which indicates the changes in the strategy of regulation of the network. For high switching parameters (*r*>1.9), we have just one regulon. Like for the toy-network, the organization of metabolic genes in a single regulon means that enzymes and transporters are constantly upregulated to avoid switching cost. A generalization of the toy-network to a large metabolism-inspired hierarchical random network, shows similar behavior as the *E. coli* core-network (see Additional file 1).

Unexpectedly, by increasing the switching parameter, *r*, the number of selected input metabolites for the optimal organism drops down for both the *E. coli* core network (Fig. 6c) and also for the hierarchical random network (Additional file 1: Figure S3c). Note that all potentially available metabolites in the environmental conditions can be taken up and converted to biomass by means of the relevant subset of the universe of metabolic reactions. Therefore, the optimal organism is able to digest a potentially available nutrient if the relevant reactions are selected in the optimal metabolic network of the organism. The obtained result shows that, for sufficiently high switching parameters, the optimal network does not choose all of the available metabolites as substrates to produce biomass. As a result, a growth rate optimized organism is not able to live under all available environmental conditions. For example, for *r*>1.9 the optimized network of *E. coli* core shows no biomass production when grow on D-glucose as the only carbon source. Note that, D-glucose supplied media considered to be the only anaerobic environment in our system setup. It seems that, it is too costly for the organism to adapt to all possible environments for high switching parameter conditions.

Considering the fact that most bacterial species, such as *E. coli*, have evolved in a highly competitive environments under strongly fluctuating nutrient conditions, our results suggest that a true bacterial generalist with the ability to survive under almost all environmental conditions will not evolve. This theoretical finding could explain the species richness of bacteria that share a common habitat [31].

### Comparison of reconstructed metabolic networks

To track the consequences of our theory, we focus on metabolic networks of bacteria, since they are considered to be metabolically optimized organisms that live in competitive environments. Some bacteria are able to reproduce in many different habitatslike *E. coli*. On the other hand, some others such as *Mycoplasma pneumoniae*, which is a parasite living inside mammalian cells, are able to reproduce just in the stable environment of the host. Now, the question is whether the properties of the metabolic networks of different bacteria living in different environmental conditions reflect their different lifestyle [32, 33]. According to the predictions of our resource constrained flux balance approach, organisms that can adapt to more diverse environments are expected to have more hub nodes of higher degree and a more pronounced bow-tie structure than organisms living under more stable conditions. To confirm this hypothesis we analyzed the structure of 143 reconstructed metabolic networks from the Model SEED database [34] with different lifestyles that are reported in the NCBI database [35] (see “Methods”).

Unfortunately, it is very difficult to get information about the different nutrient conditions under which bacterial species have evolved. We therefore classified bacteria in facultative and non-facultative (obligate) groups and compared the structural features of their metabolic networks. The reason behind this classification is that aerobic and anaerobic growth conditions are usually associated with different habitats and most facultative bacteria reproduce under both conditions whereas obligates reproduce only under anaerobic or aerobic conditions. As growth under aerobic and anaerobic conditions requires substantial readjustment of metabolic fluxes, facultative bacteria need to invest more resources for adaptation to these growth conditions than the non-facultative bacteria. The view is confirmed by observing a statistically significant difference between the structural parameters of these two groups, using the Kruskal-Wallis test [36].

**Fig. 7:**
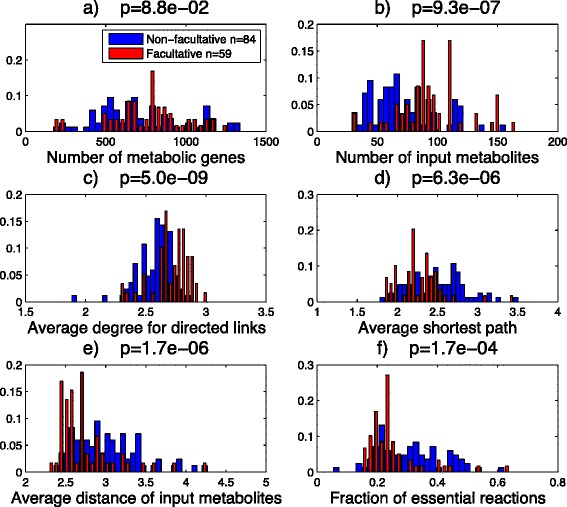
Structural parameter distributions of Reconstructed networks. Distribution of different structural parameters of 143 different species classified in two groups of facultative and non-facultative organisms with size of 59 and 84 species, respectively. The distributions of **a** number of genes used in reconstructed metabolic model, **b** number of input metabolites (transporters), **c** average in- or out-degree of the networks considering directed links, **d** average of shortest path between an input metabolite and each of biomass ingredients, **e** the average of distances between input metabolites and **f** fraction of essential reactions. p-values are calculated by the Kruskal-Wallis test

As a control observation, we found no significant difference in the distribution of the genome size between facultative and non-facultative organisms (*p*=9.4×10^−1^). Also, the difference in the distribution of the number of metabolic genes used in the reconstructed model is not significant (*p*=8.8×10^−2^, Fig. 7a). These distributions show that a fair comparison between these two groups is possible.

The facultative group has a significantly higher number of input metabolites than the non-facultative group (*p*=9.3×10^−7^ Fig. 7b), which reflects the ability of the facultative group to make use of a large variety of nutrients in comparison to non-facultative organisms. Also, the facultative group has relatively higher average degree than the non-facultative group (*p*=5.0×10^−9^, Fig. 7c). This is consistent with the results of our model, which predicts differences in the structural organization of metabolic networks for organisms having either low or high switching parameters. The lowest average degree in Fig. 7c belongs to *Onion yellows phytoplasma* which is an aerobe obligate intracellular plant parasite living in a relatively constant environment [37].

Considering *l*_*min*_ as the shortest path between any of the external metabolites and each of the metabolites that are part of the biomass reaction, the average pathway length averaged over biomass metabolites, 〈*l*_*min*_〉, is significantly smaller in the facultative group than in the non-facultative group (*p*=6.3×10^−6^ Fig. 7d). As it is mentioned previously, the number of input metabolites are on average higher in facultative organisms, which implies higher number of transporters. Many of these additional transporters are responsible for direct import of biomass metabolites, as the number of direct imports for biomass metabolites are significantly higher for facultative organisms (*p*=1.9×10^−7^ Additional file 1: Figure S7g). This fact artificially decreases the average *l*_*min*_ for facultative organisms.

As a direct measure for the bow-tie-ness of a network, we study the distance between different input metabolites or between different biomass ingredients. Since bow-tie networks have relatively more hub nodes than streamlined networks, the distances between different input or output metabolites in a bow-tie network expected to be less than streamlined networks. The results show significantly shorter distances between input metabolites or biomass metabolites for facultative organism rather than non-facultative ones. (*p*=1.7×10^−6^ and *p*=6.6×10^−5^ for input metabolites in Fig. 7e and biomass metabolites in Additional file 1: Figure S7i, respectively.) This indicates the more bow-tie structure in facultative groups. However, another direct measure of bow-tie structure is the overall closeness centralization index (OCCI) introduced by [9]. The facultative group has significantly higher OCCI than the non-facultative one (*p*=2.7×10^−6^ Additional file 1: Figure S7j), which is what we expect for more bow-tie structures.

There exists a connection between essential metabolic genes and structural features of the metabolic network. Essential reactions are defined as reactions for which their single knock-out is lethal for the organism. Only a small subset of enzymatic reactions of a bacterial metabolic network is essential for the survival in rich media [14, 15]. This fact reflects the existence of a significant amount of alternative metabolic pathways in bacteria. The existence of hub nodes in a bow-tie structured network provides more connections between different pathways rather than a streamlined network. More connection between pathways increases the possibility of using an alternative pathways. We therefore expect a significant correlation between essential metabolic genes and number and degree of hub-nodes. For example, for parasites, for which the environmental condition is stable, the structure is more linear and most of the genes are essential [32] but for more complex bacteria with the ability of living in many kinds of habitats like *E. coli*, the fraction of essential genes is low and the structure is more bow-tie like. As it is obvious from Fig. 7f, facultative organisms have significantly less essential reactions than non-facultative organisms (*p*=1.7×10^−4^). Therefore, facultative organisms have likely more alternative pathways than non-facultative organisms, which is reflected by their bow-tie structure.

## Conclusions

In summary, our results suggest that an efficiency-flexibility tradeoff can shape the structure of the metabolic networks in fluctuating environments under cellular resource constraints. This finding adds a new aspect to the evolution of microbial metabolic networks. From this point of view, the low number of precursor metabolites and thus the emergence of bow-tie structure could be the consequence of maximizing the average biomass production rate and is not necessarily the exclusive consequence of a neutral evolutionary process, as previously suggested [12].

It is currently believed that precursor metabolites are essential metabolites that connect catabolic pathways with anabolic pathways and their small number and their universality is a relict of evolution [38]. However, it can be argued that it is not necessary for the cell to rely on the existing precursors since many of them are not directly used as biomass metabolites and it is possible to bypass some of them by means of shorter alternative pathways. In fact, some of the precursor metabolites (hub nodes) are absent in the metabolic network of parasites like *Mycoplasma Pneumoniae* [32], which lives in a more stable environmental condition. Given that specialized bacteria are usually derived from generalist bacteria by genome reduction [39], it is possible that a efficiency-flexibility tradeoff is an important driving force for the universality of precursor metabolites among living cells.

Besides precursors, the efficiency-flexibility tradeoff might have narrowed down also other molecular alternatives in living cells. For example, one might ask why ATP is the only energy carrier in the cell while some other similar high-energy content molecules could also be used as energy carriers. The hydrolysis of GTP to GDP and Pi [40] is an example that can provide energy comparable to the hydrolysis of ATP to ADP and Pi. Note that changes of Helmholtz free energy during a reaction can vary considerably with pH, temperature, atmospheric pressure, and concentrations of reactants and products. Therefore, under appropriate conditions, alternative energy carriers could provide a different quantity of energy than ATP hydrolysis. The question arises why the set of possible energy carriers for metabolic reactions are narrowed down to one alternative, which is ATP. Our constrained optimization approach gives a possible answer: energy carriers are involved in different pathways that are upregulated under different conditions. By using a common energy source for all pathways the cell avoids extra investments that would be needed to generate different energy sources under different conditions.

The demand for accurate computational models is increasing due to their predictive power that could be used for designing synthetic organisms. In particular, many efforts have been made to improve FBA for achieving more realistic results [41, 42]. Our approach generalizes FBA to fluctuating environments that can provide a more realistic model for designing robust synthetic organisms living in fluctuating conditions. Considering all known enzymatic reactions (e.g. KEGG and BRENDA [43]) as the universe of reactions, one could design an optimized organism under changing environments. As the growth conditions in batch or fed-batch bioreactors can change significantly over time, our approach can be used to design organisms that can adapt to these changes.

## Methods

### Graph representation of the metabolic network

Considering the set of metabolic reactions of an organism, the graph representation of metabolic networks are not uniquely defined. For example, one definition is to consider a node for each metabolite of the reactions set and a link between any two metabolites that are on the opposite sides of each reaction. Obviously, the way a network is defined dramatically affects the structural properties of the defined network. For example, it has been shown that removing current and energy carrier metabolites like ATP, ADP, H_2_O and H ^+^ from a network increases the mean path length of the metabolic network [9, 33]. Actually, rejecting the carrier metabolites as nodes in the network would reflect a more realistic picture of the function of metabolic pathways, which is to convert an input metabolites to biomass in some sequential steps. Note that, carrier metabolites are common metabolites in many pathways that artificially increase the connectedness of the network.

We build the graph representation of metabolic networks from reconstructed metabolic models [19, 34]. We first ignore carrier metabolites the same as in [9, 33]. Each of all other metabolites are considered as a node in the network. Two copies of the same metabolite transported through the call wall exist in the metabolic models for inside and outside of the cell. The second copy that we call input metabolites are also considered to be a separate node from the original one. We then consider directed links between the substrates and products of each reaction. For revisable reactions the links are bidirectional. In this way, each reaction may define more than one link so the number of links in the network is more than the number of reactions. The explained graph representation is used in all calculations of the network parameters such as the number of selected input metabolites, number of nodes, average degree, average shortest path, and average distances.

### Simulations parameters

For the simple model simulation we choose *α*_*i*_=1, *β*_*i*_=0 and *ϕ*_0_=1. However, as it is explicit in the analytic calculations for the model in (Additional file 1: Text S1), exact values of equal catalytic rates and *ϕ*_0_ do not change the transition points.

For *E. coli* core network as the universe of metabolic reactions, we choose the average of *α* equal to 1. The average *β* is either 0 or 1 respectively for irreversible and reversible reactions. The random fluctuations of *α* and *β* are taken from a uniform distribution in [ −*σ*,*σ*], where *σ*=0 for Additional file 1: Figure S5, *σ*=0.1 for Additional file 1: Figure S6 and *σ*=0.9 for Fig. 6. We take *ϕ*_0_=100. However, the exact value of *ϕ*_0_ does not change the transition points.

### Calculation of essential reactions

To calculate the fraction of essential reactions we use the standard FBA of COBRA toolbox [44]. We put each organism in an environment containing all nutrients that the organism is able to take up. By deleting one reaction every time from the complete network, we study the ability of the organism to produce the biomass without the deleted reaction. Reactions that are necessary for producing biomass by the organism are called essential reactions and the other reactions are called non-essential reactions.

### Optimization process

Equations 1, 2, 3, 4, 5 and 6 constitutes a highly demanding linear programming problem if the number of metabolic reactions gets large. We therefore solved this problem using the SeDuMi v1.32 add-on software for MATLAB [45], as the standard routines provided by MATLAB do not have the necessary accuracy.

### Reconstructed networks selection process

From The Model SEED database [34] we just choose the complete publicly available models. To reduce the bias of statistics due to the model selection, we take into account just one of the available models of each organism. We also use the organisms that the data about their life style (facultative or non-facultative) are available on the NCBI database [35]. This leads to 143 reconstructed networks that are listed in (Additional file 2: Table S1).

### Availability of data and materials

*E. coli* Core model is taken from [29] and is publicly available at http://systemsbiology.ucsd.edu/Downloads/EcoliCore. All organisms and their living conditions considered in our statistical analysis are listed in (Additional file 2: Table S1). All metabolic models of those organisms are publicly available at the SEED database [34] in the Model SEED part.

## Ethics

Our study does not involve any humans, human data or animals.

## Additional files

Additional file 1
**Text S1.** Analytic calculation of transition points of the toy-model. Description of the Generalized random model. Supplementary Figures. (PDF 360 kb)

Additional file 2
**Table S1.** List of 143 organisms considered in the statistical analysis of bacterial metabolic networks and related informations. (XLSX 42 kb)
