# Supplementary material for: Resource constrained flux balance analysis predicts selective pressure on the global structure of metabolic networks
Source: BMC Syst Biol. 2015 Nov 23;9:88. doi: 10.1186/s12918-015-0232-5 (PMC4657269; doi:10.1186/s12918-015-0232-5)
Supplement: Additional file 1 — Text S1. Analytic calculation of transition points of the toy-model. Description of the Generalized random model. Supplementary Figures. (PDF 360 kb) [file 12918_2015_232_MOESM1_ESM.pdf]

Supporting Information: Resource Constrained Flux Balance Analysis predicts Selective Pressure on the Global Structure of Metabolic Networks

Nima Abedpour and Markus Kollmann

Analytic calculations of transition points for the toy-network

To calculate transition points for the toy-network, we look for an optimal network that maximizes the flux of biomass reaction for a given switching parameter r. We first consider all possible structures and strategies of regulation that fulfill the constrains in Eq. 1-5 of the main text. Afterwards, by comparing the maximum biomass flux of those different structures and strategies with the same maximum resource investments of φ_0, we are able to find the optimal structure and strategy of regulation for the optimal network that maximizes the growth rate.

For each environmental condition there are two independent pathways that satisfy the constraint of Eq. 1 of the main text. For example, Fig. S1 shows the two independent pathways, $P1$ and $P2$, for the first environmental condition containing nutrient $S1$. Using these two pathways, two possible structures for the network can be considered. Note that choosing $P1$ for one environmental condition and $P2$ for the other environmental condition has no benefit since this would increase the invested metabolic resources of each environment and the investment of the switching conditions at the same time.

In the first transition, considering ν_{max} as the maximum flux of the pathways, the total investment of the enzymatic resources of $P1$ is $5 \times \nu_{max}$ since the pathway contains 5 reactions and the invested resources for the switching condition is $r \times 2 \times 4 \times \nu_{max}$ related to 2×4 unshared reactions of the pathways of two different environmental conditions. Similarly, the total investment of enzymatic resources of $P2$ is $6 \times \nu_{max}$ and the invested resources for the switching condition is $r \times 2 \times 2 \times \nu_{max}$. So the resource constraint of Eq. 4 of the main text related to each pathway is

$$5 \times \nu^1_{max} + r \times 2 \times 4 \times \nu^1_{max} < \varphi_0 \quad (1)$$
$$6 \times \nu^2_{max} + r \times 2 \times 2 \times \nu^2_{max} < \varphi_0$$

where ν^1_{max} and ν^2_{max} are the optimal fluxes using pathway $P1$ and $P2$ respectively. So the maximum fluxes in both cases are proportional to φ_0 and are functions of r:

$$\nu^1_{max} = \frac{\varphi_0}{5+8r} \qquad (2)$$
$$\nu^2_{max} = \frac{\varphi_0}{6+4r}$$

Now if ν^1_{max} has a grater value, $P1$ would be the optimal pathway and if ν^2_{max} is larger, $P2$ would be optimal pathway. The transition, which depend on r, occurs when the right hand sides of Eqs. 2 become equal that happens at $r = 0.25$. For $r < 0.25$, $P1$ has a larger flux and for $r > 0.25$, $P2$ has a larger flux. In this way, by changing r we change the cost of switching investments and change the structure of the optimized network between two networks of Fig. 3a and Fig. 3b of the main text.

For the second transition, the structure of the pathways of the network are the same (like $P2$) but with different regulations. In the first kind of regulation, which is up- and downregulating the needed enzymes, the total investment of the metabolic resources is $6 \times \nu_{max}$ and the invested resources for the switching condition is $r \times 2 \times 2 \times \nu_{max}$. But in the second kind of regulation, which is constantly upregulating all enzymes, the total investment of the metabolic resources is $8 \times \nu_{max}$ and the cost of the investment is 0 since in this strategy all of the enzymes are expressed simultaneously and there is no cost for regulation in switching condition. So the resource constraint of Eq. 4 of the main text is like

$$6 \times \nu^1_{max} + r \times 2 \times 2 \times \nu^1_{max} < \varphi_0 \qquad (3)$$
$$8 \times \nu^2_{max} + 0 < \varphi_0$$

where ν^1_{max} and ν^2_{max} are the optimal fluxes using first and second regulation strategy respectively. So the maximum fluxes in both case are proportional to φ_0 and are a function of r:

$$\nu^1_{max} = \frac{\varphi_0}{6+4r} \qquad (4)$$
$$\nu^2_{max} = \frac{\varphi_0}{8}$$

Now if ν^1_{max} has a greater value, first regulation strategy is the optimal one and if ν^2_{max} is larger the second one is optimal strategy. The transition, which depends on r, occurs when the right hand sides of both equations in Eq. 4 become equal that is at $r = 0.5$. For $r < 0.5$ the first strategy would have larger flux and for $r > 0.5$ the second one. In this way, by changing r we change the cost of investment in resource constraint and change the strategy of regulation for the optimized network. These results are in agreement with computational results of the main text.

Generalized random network model

To generalize the simple toy-model, we consider a hierarchical random network as the universe of reactions as shown in Fig. S2. The first layer of the network contains input metabolites and the last layer contains biomass contents. Metabolites in the intermediate levels are intermediate metabolites of the pathways that convert input metabolites to biomass. The structural properties of the random networks is shown in Fig. S3 for different switching parameters, r. Here, the considered universe of reactions has 3 intermediate levels with 15 metabolites for each level, 7 input metabolites and 7 metabolites as the biomass content. Each level is connected to the next level by means of 20 random reactions. To increase the complexity of the network, 20 additional reactions are also considered randomly between any two metabolites of the network.

As it is shown in Fig. S3, any changes in the switching parameter change the structural parameters of the network. The behavior of structural parameters, like growth rate; ASP, number of regulons and number of selected input metabolites, are similar to the results of our simple toy-model and *E.coli* core metabolic network. However, here we see almost continuous transitions due to an averaging over 400 realizations. Sharp changes can be observed in the results of a single realization such as Fig. S4.

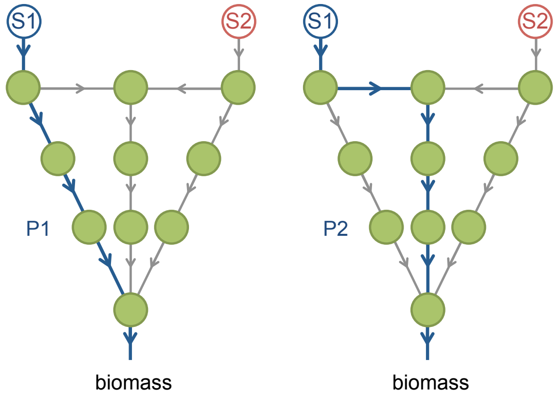

Figure S1: Alternative pathways. Bold arrows represent two possible pathways, which preserved flux balance constraint, in presence of substrate $S1$ as the environmental condition.

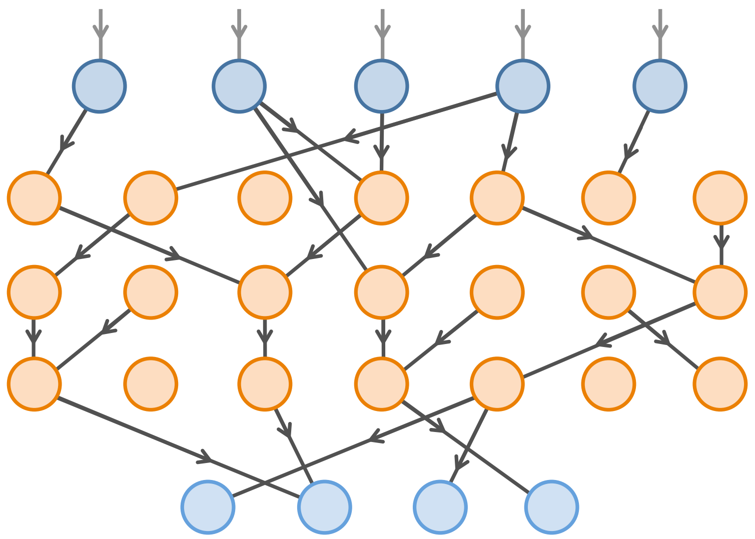

Figure S2: Generalized random network structure. Metabolites in the first layer (dark blue) are the input metabolites and in the last layer (blue) are the biomass content. Metabolites of the other levels (orange) are the intermediate metabolite of the intermediate reactions that can be used to convert input metabolites to biomass.

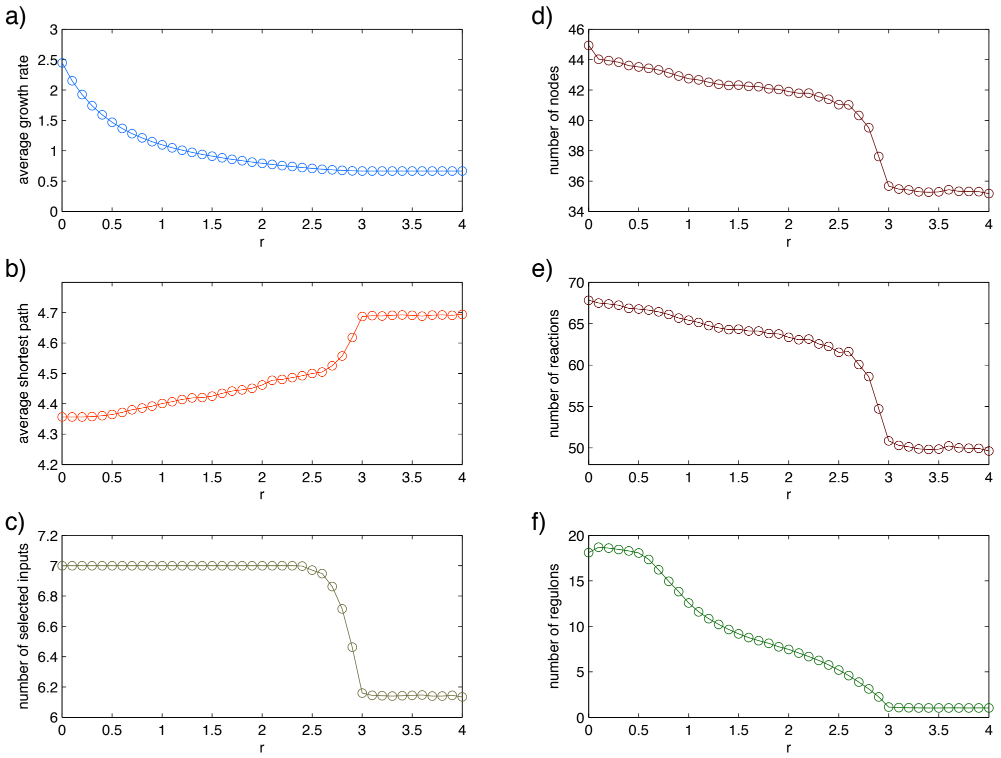

Figure S3: Structural parameters for the generalized random model. The considered network has 59 metabolites and 108 reactions as the universe of metabolic networks (3 intermediate levels with 15 metabolites, 7 input metabolites and 7 metabolites in biomass). We average the quantities over 400 realizations. a) average growth rate vs. r. b) average shortest path from an input metabolite to each of the biomass contents for the optimized network vs. r. c) number of selected input metabolites after optimization process vs. r. d) number of nodes of the optimized network vs. r e) number of reactions of the optimized network vs. r. f) number of regulons vs. r.

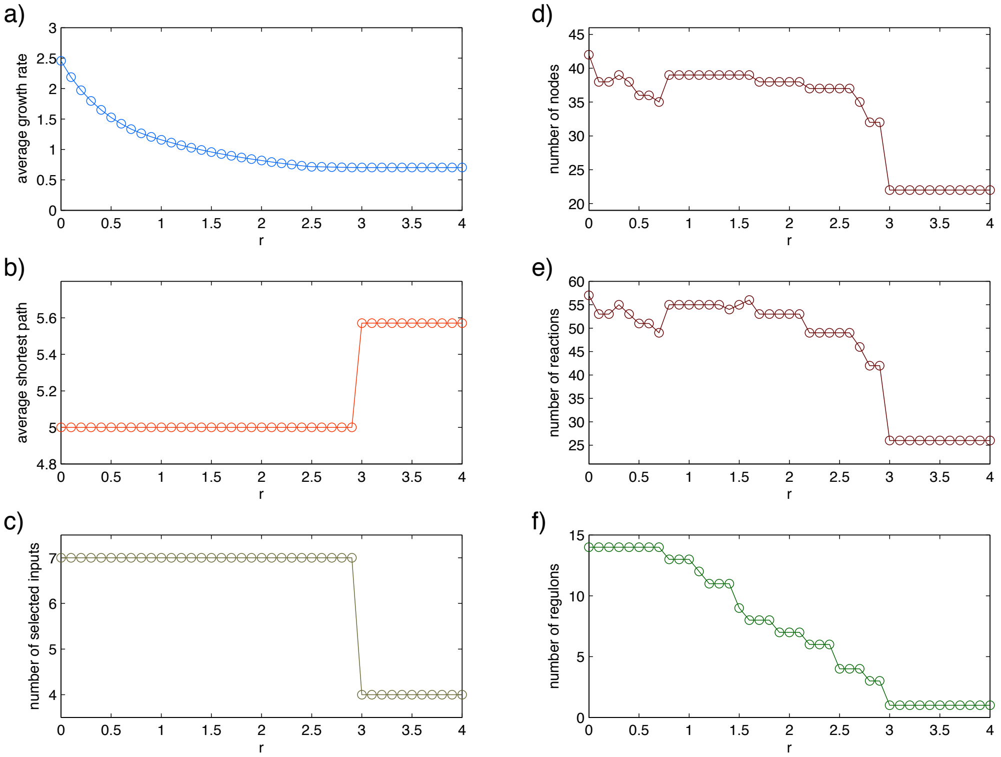

Figure S4: Structural parameters for a single realization of the generalized random model. Considered network has 3 intermediate levels with 15 metabolites, 7 input metabolites and 7 metabolites in biomass. It has also 108 random reactions that connect different levels. a) average growth rate vs. r. b) average shortest path from an input metabolite to each of the biomass contents for the optimized network vs. r. c) number of selected input metabolites after optimization process vs. r. d) number of nodes of the optimized network vs. r e) number of reactions of the optimized network vs. r and f) number of regulons vs. r.

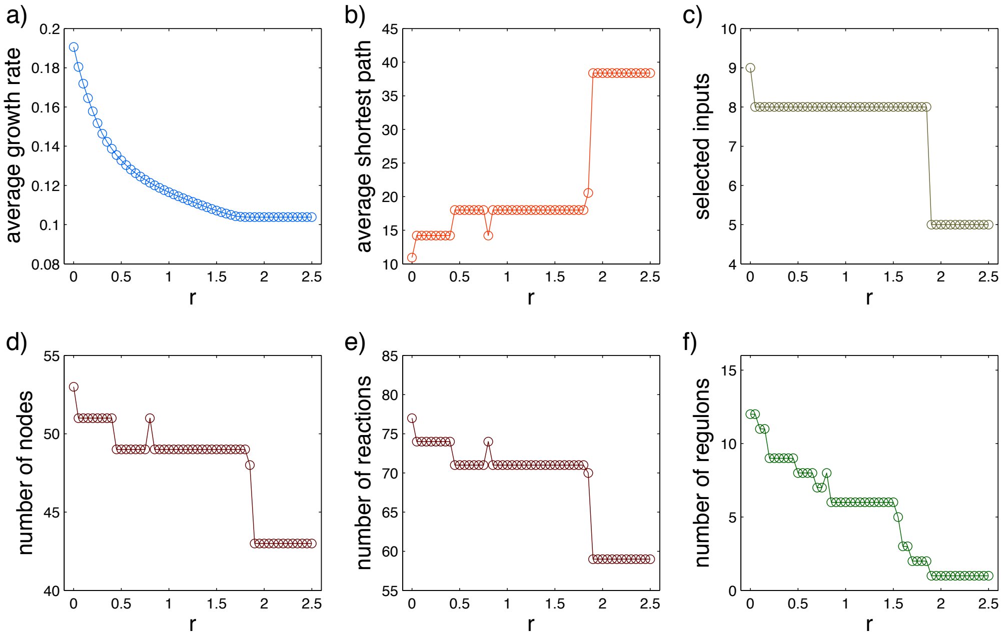

Figure S5: Structural parameters for the *E. coli* core metabolic network. Here, α and β values are the same. 5 different environmental conditions that represent different carbon sources with 2 to 6 carbon atoms is used. a) average growth rate vs. r. b) average shortest path from an input metabolite to each of the biomass contents for the optimized network vs. r. c) number of selected input metabolites after optimization process vs. r. d) number of nodes of the optimized network vs. r e) number of reactions of the optimized network vs. r. f) number of regulons vs. r.

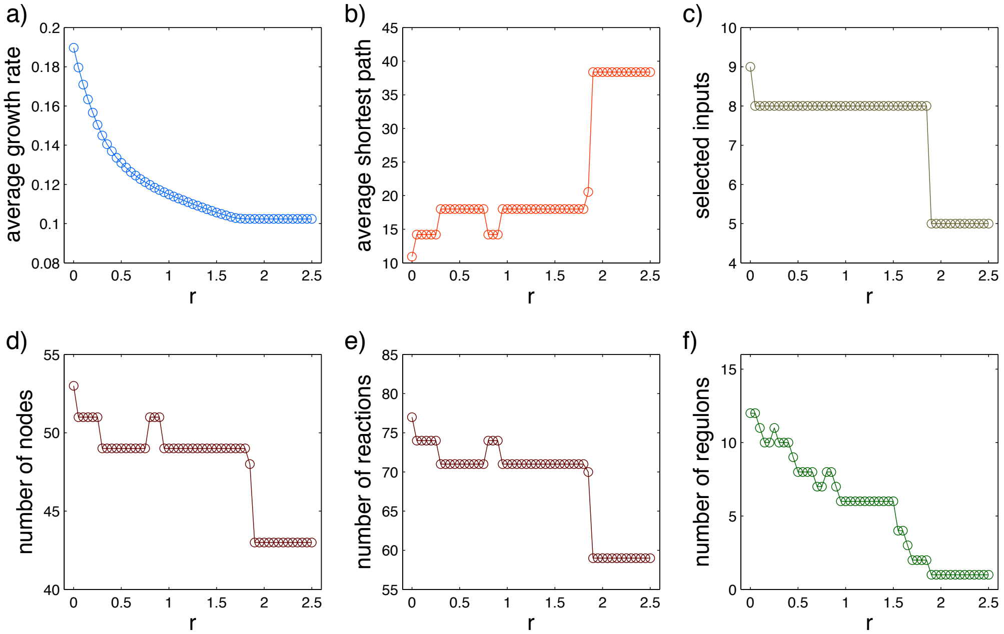

Figure S6: Structural parameters for the *E. coli* core metabolic network. A single realization of α and β with 10 percent fluctuations is used. 5 different environmental conditions that represent different carbon sources with 2 to 6 carbon atoms is used. a) average growth rate vs. r. b) average shortest path from an input metabolite to each of the biomass contents for the optimized network vs. r. c) number of selected input metabolites after optimization process vs. r. d) number of nodes of the optimized network vs. r e) number of reactions of the optimized network vs. r. f) number of regulons vs. r.

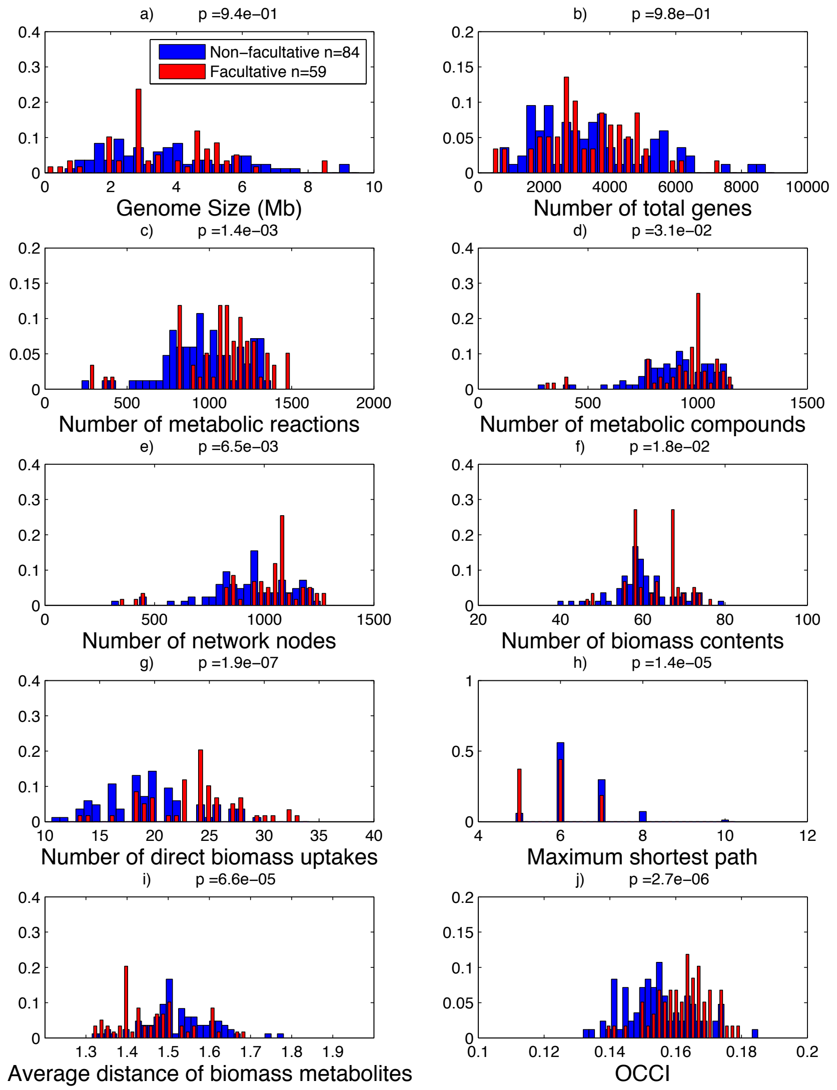

Figure S7: Distribution of different structural parameters of 143 different organisms classified in two groups of Facultative and non-Facultative organisms with the size of 59 and 84 respectively. Distributions of a) genome size in Mb, b) number of total genes, c) number of reactions in reconstructed metabolic model, d) number of compounds in reconstructed metabolic model, e) number of network nodes, f) number of biomass metabolites, g) number of direct links from an input metabolite to biomass, h) maximum length of shortest path, i) the average of distances between biomass metabolites and j) overall closeness centralization index (OCCI). p-values are calculated by the Kruskal-Wallis test.
